# Surface plasma with an inkjet-printed patterned electrode for low-temperature applications

**DOI:** 10.1038/s41598-021-91720-3

**Published:** 2021-06-09

**Authors:** Jinwoo Kim, Sanghoo Park, Wonho Choe

**Affiliations:** 1 Department of Physics, Korea Advanced Institute of Science and Technology (KAIST), 291 Daehak-ro, Yuseong-gu, Daejeon, 34141 Republic of Korea; 2 Institute of Plasma Technology, Korea Institute of Fusion Energy (KFE), 37 Dongjangsan-ro, Gunsan, Jeollabuk-do 54004 Republic of Korea; 3 Department of Nuclear and Quantum Engineering, KAIST, 291 Daehak-ro, Yuseong-gu, Daejeon, 34141 Republic of Korea

**Keywords:** Plasma physics, Chemical biology

## Abstract

The global health crisis caused by the recent pandemic has led to increasing social demand for ‘new normal’ sanitizing and disinfecting facilities to fit our ‘new normal’ lives. Here, we introduce an inkjet-printed, thin-film plasma source applicable to dry disinfection processes. In contrast to conventional plasma reactors, the merits of plasma produced on a film include disposability, cost-effectiveness, and applicability to high-dimensional objects such as the human body. The developed flexible plasma film can be applied to a wide variety of shapes via origami—remaining plasma stable even when bent. However, electrode degradation has been a practical issue in the long-term operation of inkjet-printed plasma sources, which is troublesome from application perspectives. We focus on making the inkjet-printed electrode more plasma stress-resistant, thereby increasing its lifespan from a few minutes to two hours of continuous operation with optimal inkjet printing and passivation, thus increasing the practicality of the source. Considering the fact that ozone and nitrogen oxides are selectively produced by plasma, we implement a disposable pouch-type plasma source and examine its usefulness in extending the shelf life of food.

## Introduction

As the novel coronavirus (COVID-19) has rapidly advanced around the world, infectious disease management ranging from personal hygiene to social quarantine has recently gained increasing importance. A growing number of researchers have partly turned to apply nontraditional approaches to create ‘new normal’ protective measures to prepare for the post-COVID-19 era. One emerging technology is cold plasma due to its effectiveness in bacterial, viral, and fungal treatments^1^.

Plasma contains energetic charged particles and reactive species, a strong electric field, UV radiation, etc., which have significant physicochemical effects on biomaterials^2^. In contrast to conventional plasmas under vacuum, atmospheric pressure plasma is usable in open space in ambient air, of which unique features make it easy to apply in everyday life. Thus, among the various types of plasmas, cold plasma generated at atmospheric pressure has led to new developments in material science and engineering fields related to food, agriculture^3–5^, and biomedicine^6^.

Since ‘plasma sterilization’ research started in the 1990s^7–9^, remarkable results have been achieved, ranging from the inactivation of bacteria and fungi on food^10–15^ to the disinfection of livestock^16,17^. Having experienced that atmospheric pressure plasmas merit in wide scientific and industrial ranges, the needs of flexible plasma sources have been discussed. The deformable feature of a flexible plasma source allows direct plasma contact with high-dimensional target surfaces, and this advantage increases the applicability of the source; some examples include wearable plasma devices that can be applied with self-sterilizing clothes and face masks, bandages for wound healing, and plasma-assisted food packaging for maintaining food quality and preventing spoilage. Some kinds of flexible plasma apparatuses have been introduced as forms of multiple electrode arrays^18^, parallel copper wires in water-filled PTFE tubes^19^, modified coaxial wires^20^, and metalized paper sheets^21^. Most apparatuses rely on surface dielectric barrier discharge (DBD), as its configuration and material are mechanically optimal to add flexibility.

**Figure 1:**
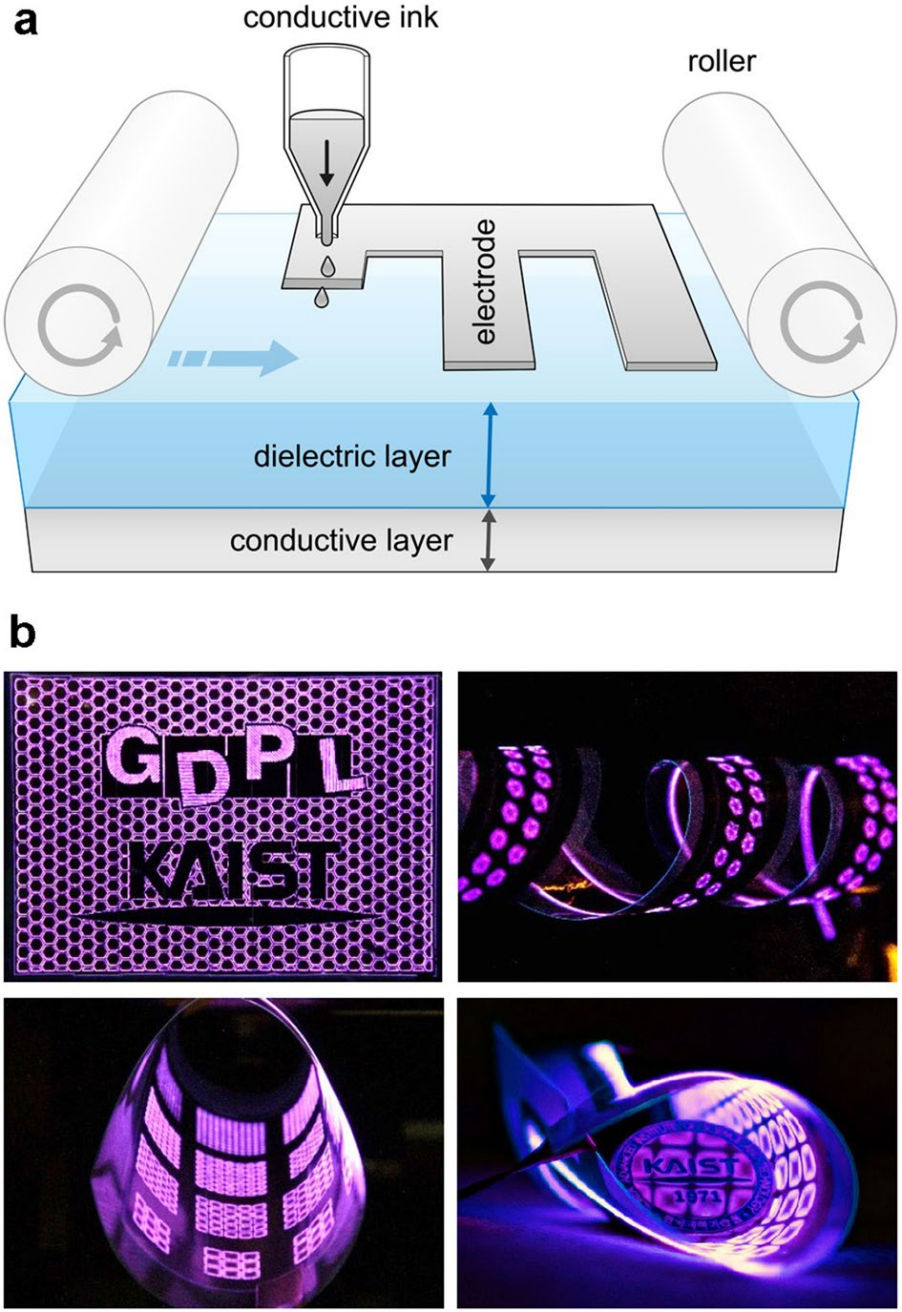
(**a**) Schematic illustration of inkjet printing used to form a thin-film electrode on a flexible dielectric barrier discharge source. The cross-sectional view of the FXDBD source shows two conductive layers and a dielectric barrier film (PET). (**b**) Examples of FXDBD with different inkjet-printed, patterned electrodes operated in ambient air to demonstrate the flexibility of electrode formation (i.e., a large variety of electrode shapes and sizes) and the flexibility of the substrate.

Here, we report the first approach for fabricating flexible dielectric barrier discharge (FXDBD) sources through inkjet printing. There are several kinds of methods for forming a conductive thin layer on a film substrate: drawing^22,23^, stamp tapping^24^, silk-screening^25,26^, etc. In particular, inkjet printing is the most attractive way to fabricate an FXDBD source; the corresponding electrodes, one or more, can be formed on a thin-film dielectric substrate using a conventional inkjet printer with electrically conductive ink (Fig. 1a). This simple setup provides fast and cost-effective manufacturing and accommodates different designs in lab-scale uses^27,28^. We observed that the physicochemical stress caused by the plasma degrades the bare-inkjet-printed electrode and affects plasma properties over time, which is critical in plasma applications. Thus, some efforts have been dedicated to overcoming the degradation of inkjet-printed electrodes. Furthermore, we investigate the chemical characteristics of FXDBD, which can be informative in future applications. Our findings reveal that FXDBD can be selectively operated in ozone-dominant or ozone-free mode, with potential merit in various material science and engineering fields. Finally, as a feasible example, our FXDBD source is applied to a food packaging pouch to lengthen the shelf life of blueberries.

## Results and discussion

### Inkjet-printed FXDBD

In this study, commercial silver nanoparticle ink (Mitsubishi Paper Mill) and an office printer (Brother, DCP-T300) were employed. Since printing was performed using a commercial office printer, the maximum size of FXDBD that can be currently produced in the A4 paper size; however, enlargement of the production size is viable by replacing the printer. Care was taken with inkjet printing, as ink properties such as viscosity, surface tension, volatility, and particle size are critical in the jetting process from the nozzle. In particular, we used a printer with a piezoelectric-type nozzle to prevent the thermal deterioration of the ink in printing processes. Prior to plasma experiments, we determined the optimal printing conditions to maximize the density of ink drops and consequent conductivity of the printed ink, such as humidity and temperature of the room where printing was performed (20–40 °C, 40% humidity). We used the printing media which has a PET substrate with resin on it (Mitsubishi Paper Mill). A porous chemical (resin) layer on it effectively absorbs ink and prevents smearing, improve the overall inkjet printing performance on the film. The electrical resistivity and thickness of the inkjet-printed electrode were measured using a 4-point probe method (Keithley 6221 current source combined with a Keithley 2182a nano voltmeter) and Tencor P-2 long-scan profiler, respectively.

The designed electrode was printed onto the film based on black vector images drawn by commercial software on a computer. We chose the electrode pattern size in such a way that the power dissipated to the plasma became maximum at the same applied voltage. The FXDBD source under experiment consisted of 10 × 10 square grids with an area of 7 mm × 7 mm each grid. An additional copper sheet attached to one side of the FXDBD source was used as a powered electrode by connecting it to the power system. The power delivered to the FXDBD was measured in the range of 5 to 45 W depending on the input voltage via the charge–voltage (Q–V) Lissajous method. Even when bent with a 2 mm bending radius, the FXDBD source exhibited uniform and stable discharge (Fig. 1). The discharge power was compared with different bending radii ranging from 50 to 2 mm using the same FXDBD source. We found that both current and voltage measured at the electrode remained the same as 4 A_max_, 6.5 V_pp_ (15 W) regardless of the bending radii. Based on such high deformability, a three-dimensional and large-area plasma generator with a complicated shape can be implemented, as demonstrated via origami in Fig. 1b.

### Critical issues for inkjet-printed electrodes

**Figure 2:**
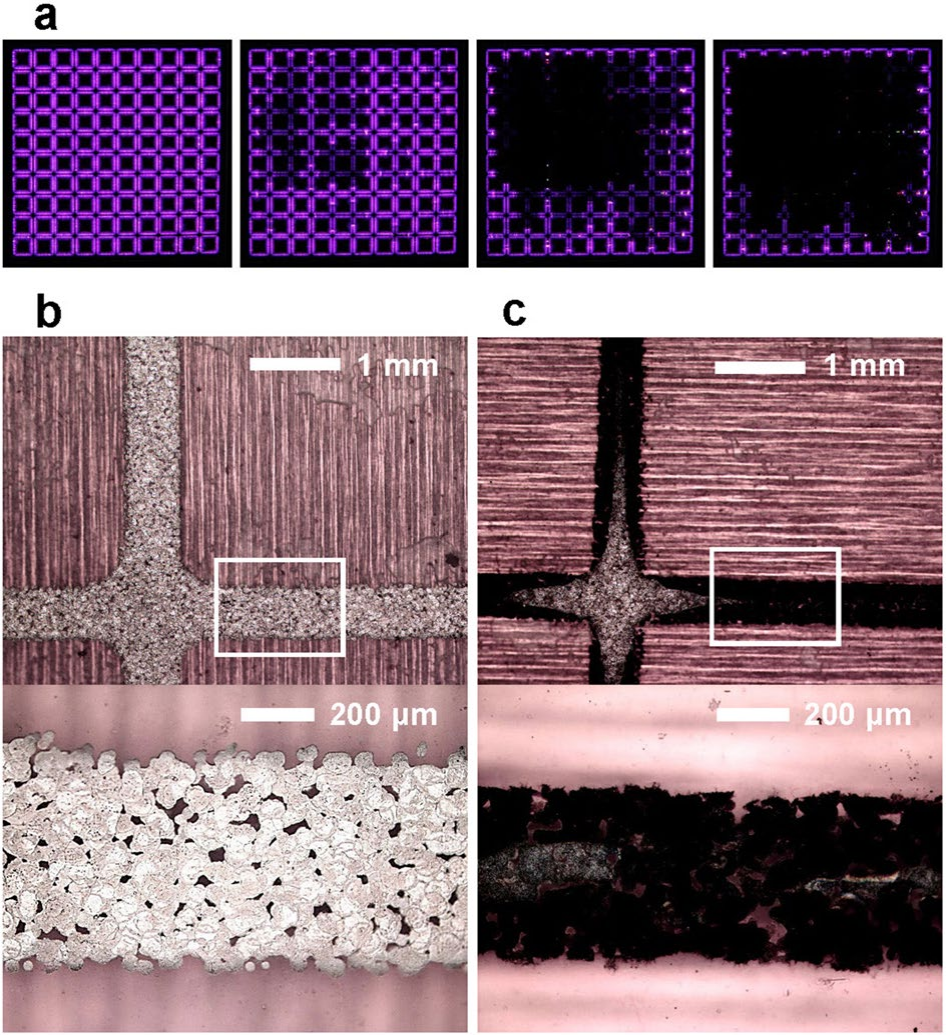
(**a**) Degradation of plasma emissions from FXDBD with a 5 kVpp sinusoidal voltage at 3 kHz. The time interval between images is 1 min. Note that the plasma in the leftmost image is shown 3 min after the operation began. Microscope-obtained photographs of an inkjet-printed electrode on (**b**) unused and (**c**) used FXDBD sheets, respectively. The bottom images show magnified photographs of the upper images, with the corresponding areas indicated by the white boxes.

In general, physicochemical stress is unavoidable for bare-metal electrodes directly contacting reactive plasma, resulting in electrode corrosion over time. Notably, we experienced the degradation of plasma performance during long-term operation, as the bright-purple plasma began to evanesce and finally disappeared entirely from the electrode (Fig. 2a). In the unused case, the droplets of silver nanoparticle ink merged well with neighboring droplets, forming an electrically good connection. However, after 6 min of plasma operation, the printed electrode was permanently destroyed; we observed changes in the electrode shape and color (Fig. 2b,c). The material analysis of inkjet-printed electrode using the energy-dispersive X-ray spectroscopy (EDS) indicated that the atomic percent of oxygen and carbon were increased from 13.5% to 34.1% and 4.4% to 8.1%, respectively, due to the 6 min plasma operation. The limited durability of the FXDBD may be problematic, particularly for applications in which high plasma reliability and reproducibility are required.

There are two major reasons for this limitation. The first reason is the chemical reaction between the electrode and oxidative chemical species, such as ozone. The increases in the gas temperature and oxidant concentration due to plasma gradually accelerate the oxidation of the inkjet-printed electrode, thereby resulting in ohmic dissipation. The second reason is ‘physical sputtering’ due to collisions between energetic particles and the electrode surface. Since this physical process largely occurs at the electrode edge in contact with the plasma, it progresses from the edge to the inside of the electrode, resulting in significant electrode damage^29^. Particularly, such permanent damage is more severe in the case of FXDBD than in other cases because the thin-film electrode is particularly delicate and can be completely disconnected.

### Fabrication of plasma stress-resistant FXDBD

Two methods were attempted to increase the durability of the inkjet-printed electrode; these methods are good for not only extending the FXDBD lifespan but also improving the power efficiency. Note that the lifespan was defined as the plasma-on period in relation to power dissipation—the period from the onset of discharge to the time when the discharge power decreased rapidly.

**Figure 3:**
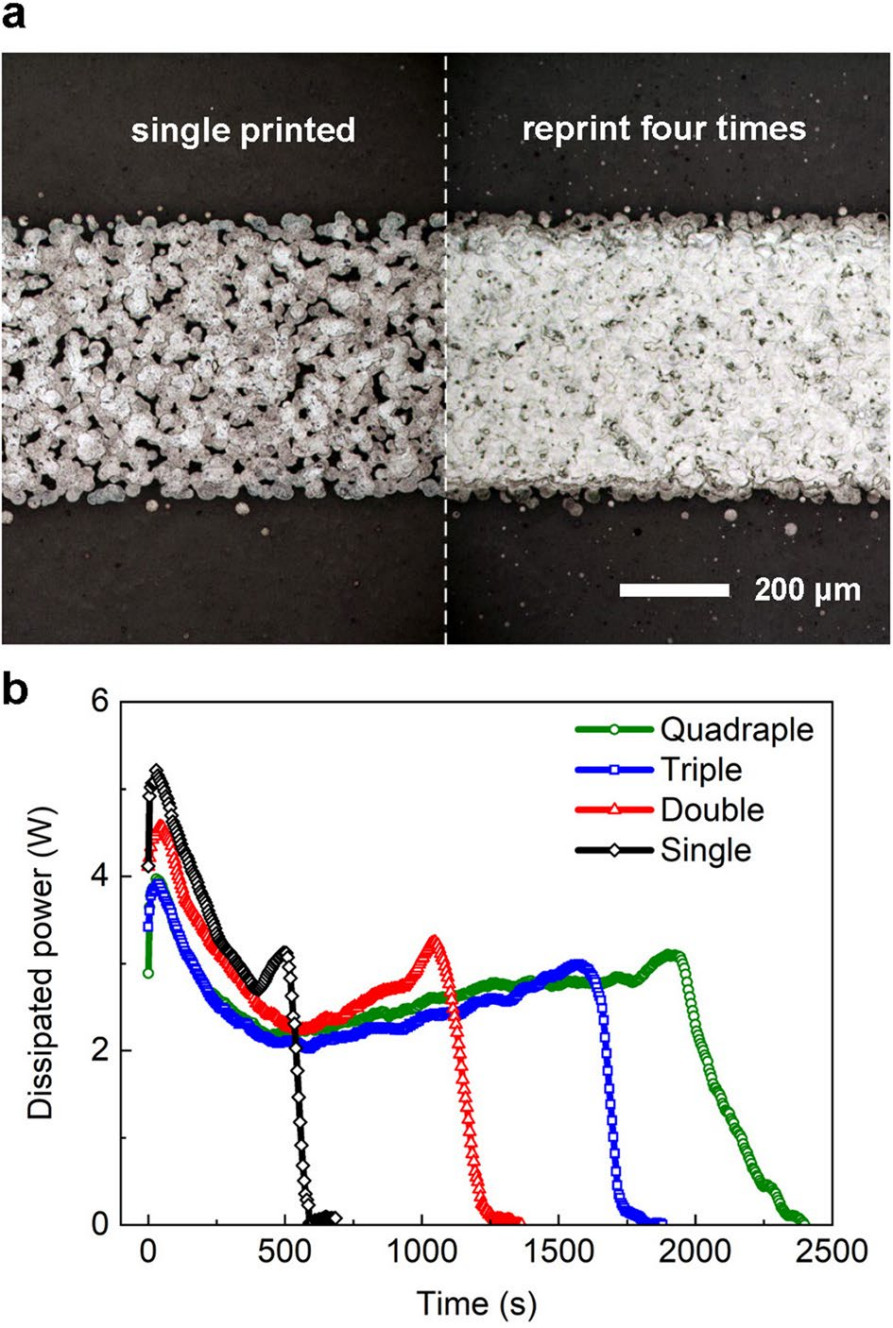
(**a**) Optical images of single- and quadruple-printed electrodes. (**b**) Power is delivered to the plasma as a function of time to show the lifespan of FXDBD based on the number of printings.

Figure 3a shows a comparison of microscope images of the electrode printed once and four times. The image clearly shows that the ink ejected from the nozzle takes the form of dots that connect to form a continuous conductive link. It also shows the micro-gaps created by the irregular distribution of ink and air bubbles inside the electrode. Therefore, a single-printed electrode displays a sparse density of the ink, while these gaps are completely covered by repetitive printing. This trend also appears in the electrode thickness and resistivity measurement results. The electrode thickness was not linearly proportional to the number of printing, and resistivity was dramatically reduced due to the gap filling effect by repetitive printing. With an increasing number of prints from one to four, the electrode thickness increased to 310, 365, 520, and 890 nm, and the corresponding resistivity decreased to 300, 29.4, 16.3, and 19.0 nΩ·m at 289 K, respectively. Also, power-consuming micro discharges that may occur inside sparse electrodes were largely suppressed, and the overall delivered power gradually decreased with an increasing number of printings. Figure 3b shows the power delivered to the plasma as a function of the discharge time with a different number of prints under the same operating conditions with a 5 kV_pp_ sinusoidal voltage waveform at 3 kHz.

The lifespan of FXDBD sources with 1–4 printings were approximately 500, 1100, 1550, and 1900s, respectively. Although the electrode thickness was increased by 18%, the lifetime increased by more than twice for the double-printed FXDBD. However, in the fourfold case, the discharge lifetime increased by only 22% over that in the threefold case, whereas the thickness increased by 71%. Based on the aforementioned relationship between the lifetime and electrode thickness of the FXDBD, the efficacy of micro discharge suppression by multi printing is apparent. During micro discharges that occur in gaps, plasma faces all the micro edges of each ink drop, and the plasma-facing area is significantly increased compared to the electrode volume. Thus, gap-filling prevents both physical sputtering and chemical oxidation over the entire electrode surface, thus improving the physicochemical resistance of the inkjet-printed electrode.

**Figure 4:**
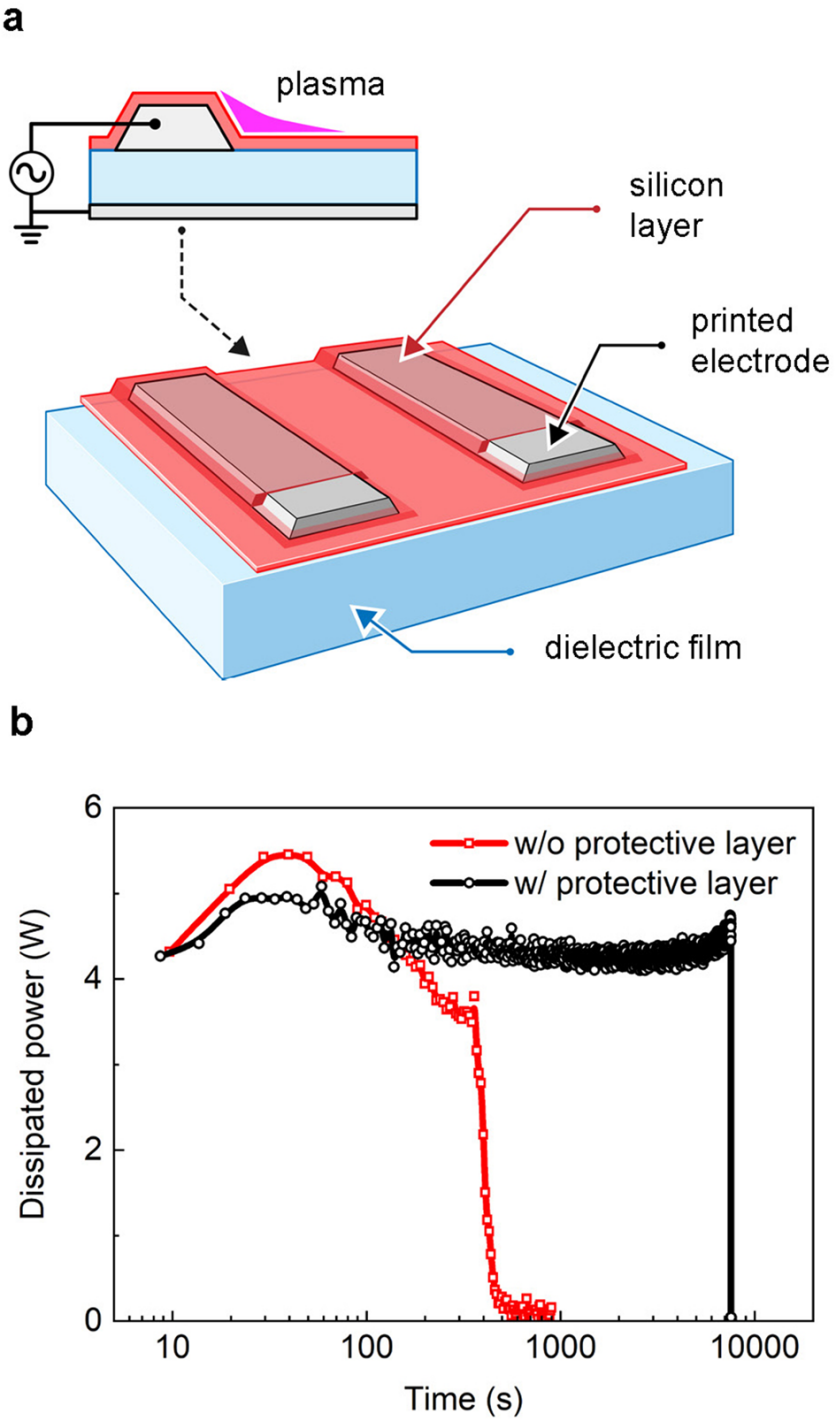
(**a**) Schematic view of a silicon-coated FXDBD source. (**b**) Dissipated power of FXDBD with and without electrode passivation.

One of the options for improving the FXDBD lifespan is electrode passivation, which physically separates the electrode from reactive plasma species. Figure 4a shows the structure of the FXDBD source with a passivation layer; the bare inkjet-printed electrode was coated with silicon, of which the thickness was in the range of 20–50 µm. To prevent discharge from occurring in the gap between the electrode and protective layer, liquid silicon was uniformly applied to the entire surface of the FXDBD. In doing so, plasma was produced on the passivation layer without interacting with the electrode. Silicon was chosen as a material for the protective layer because it is not only widely used in various industries, but also flexible, inexpensive, and transparent enough to check the extent of damage to the electrode. The composition of the silicone used in the experiment was 30% silicone polymer, 30% dimethyl ether, 25% toluene, and 15% acetone.

Figure 4b shows the change in the power delivered to the plasma over time with and without the protective layer. An uncoated, single-printed FXDBD ran for 8.3 min, and the lifespan exceeded 120 min with the protective layer, a value 14 times larger than that in the former case. The results suggest that this approach is valid for increasing the electrode life from both physical and chemical perspectives. First, a physical buffer between the electrode and plasma is created, thereby reducing electrode damage from plasma stress. Second, a chemical barrier against oxidizing plasma species is formed. However, the protective layer is not permanent because it was irreversibly damaged by the plasma over time, eventually resulting in electrode dissipation.

Moreover, we observed an increase in the electrical (power) stability of the FXDBD with the addition of electrode passivation, namely, a narrow variation in dissipated power with time (Fig. 4b). Both sources with and without the protective layer displayed initial power dissipation at approximately 4.2 W. However, dissipation power decreased from 5.5 to 3.4 W over time in the bare electrode case, whereas a slight decrease in dissipated power from 4.9 to 4.2 W was observed over time in the presence of the protective layer. Because the input power is directly associated with the uniform generation of reactive species, as discussed below, it is an important factor in ensuring the consistency and reliability of a plasma source from application perspectives.

### Gaseous chemical characteristics of FXDBD

**Figure 5:**
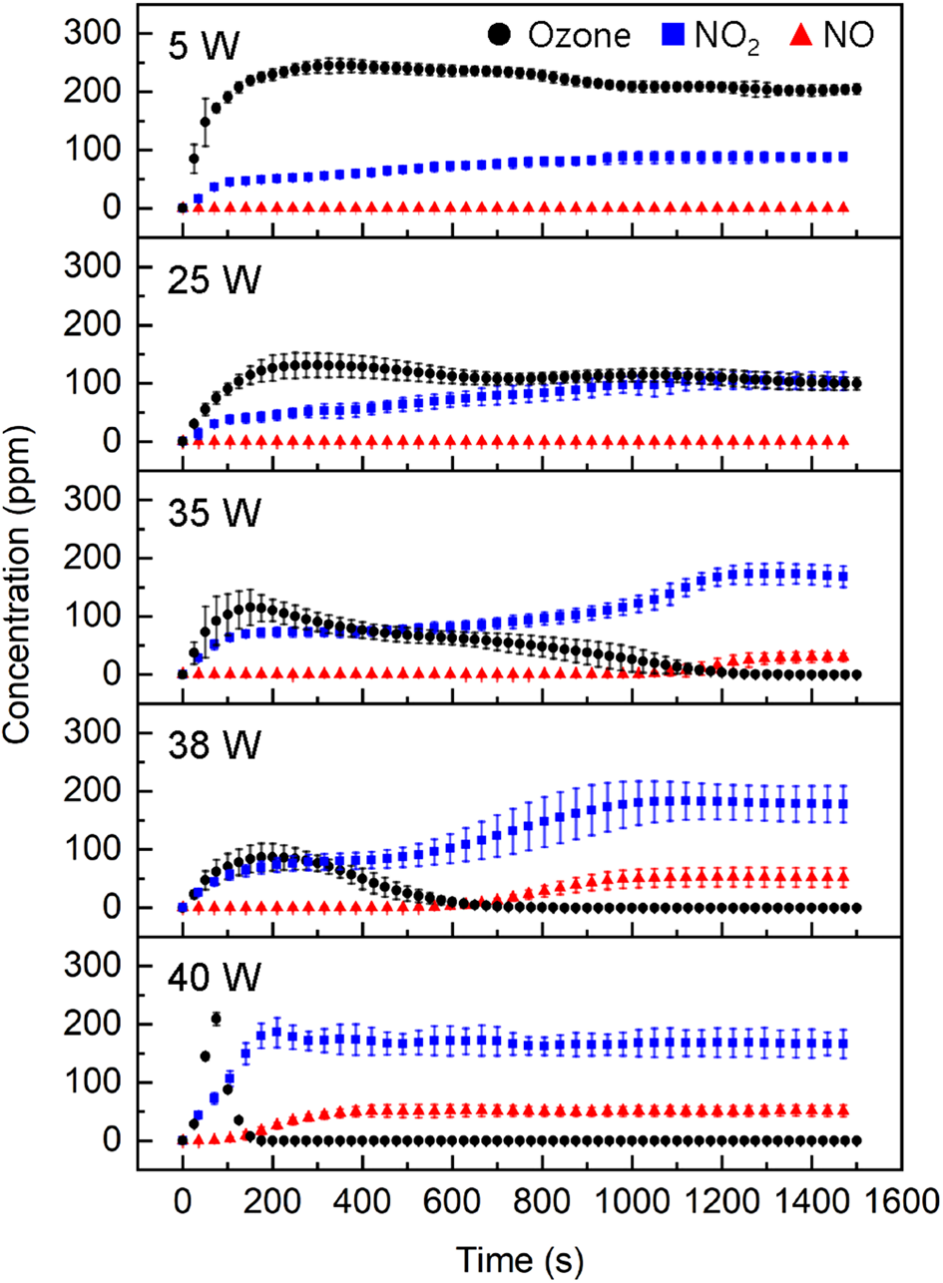
Concentrations of O_3_, NO_2_, and NO produced by FXDBD at different driving powers. Data are shown as the mean and standard deviation from three independent measurements.

Characterization of the plasma source is a prerequisite for satisfactory application. Thus, we investigated the relationship between reactive oxygen and nitrogen species (RONS) production in FXDBD and plasma-dissipated power. Hereinafter, the FXDBD source was operated using a 30 kHz bipolar square wave voltage source in the discharge power range of 5–45 W. Figure 5 shows the concentrations of O_3_, NO, and NO_2_ generated by an FXDBD source inside the chamber with different discharge power. In the 5 W case, O_3_ was the major chemical that appeared first, and its concentration rapidly reached a plateau. Simultaneously, NO_2_ increased gradually and reached a steady level after 1200 s, whereas the NO concentration remained below the detection limit (0.05 ppm). However, at high discharge power, O_3_ depletion occurred due to the quenching reaction of O atoms with NO and NO_x_^30^. As the input power increased to 35 W, ozone, the concentration of which peaked near 200 s, gradually decreased; the ozone concentration fell below the detection limit at approximately 1200 s. Accordingly, a clear mode transition from O_3_ to NO_2_ was apparently observed at approximately 500 s; the high discharge power case showed relatively rapid crossover between the NO_2_ and O_3_ concentrations. At 40 W, there was no O_3_ after 200 s, and the concentrations of NO and NO_2_ rapidly reached plateaus of 50 and 170 ppm, respectively. As the input power was increased, the concentration of vibrationally excited nitrogen molecules also increased, which actively react with O atoms, thereby decreasing O_3_ production^30^. Accordingly, it was concluded that O_3_ and NO have an interdependent relation in FXDBD, and this relation is quite similar to that for conventional surface DBDs^31,32^. As shown, the chemical production of O_3_, NO, and NO_2_ is also controllable in FXDBD depending on the operation conditions, thus highlighting the potential for expanding the utilization of the FXDBD.

### Feasibility test for the FXDBD pouch

**Figure 6:**
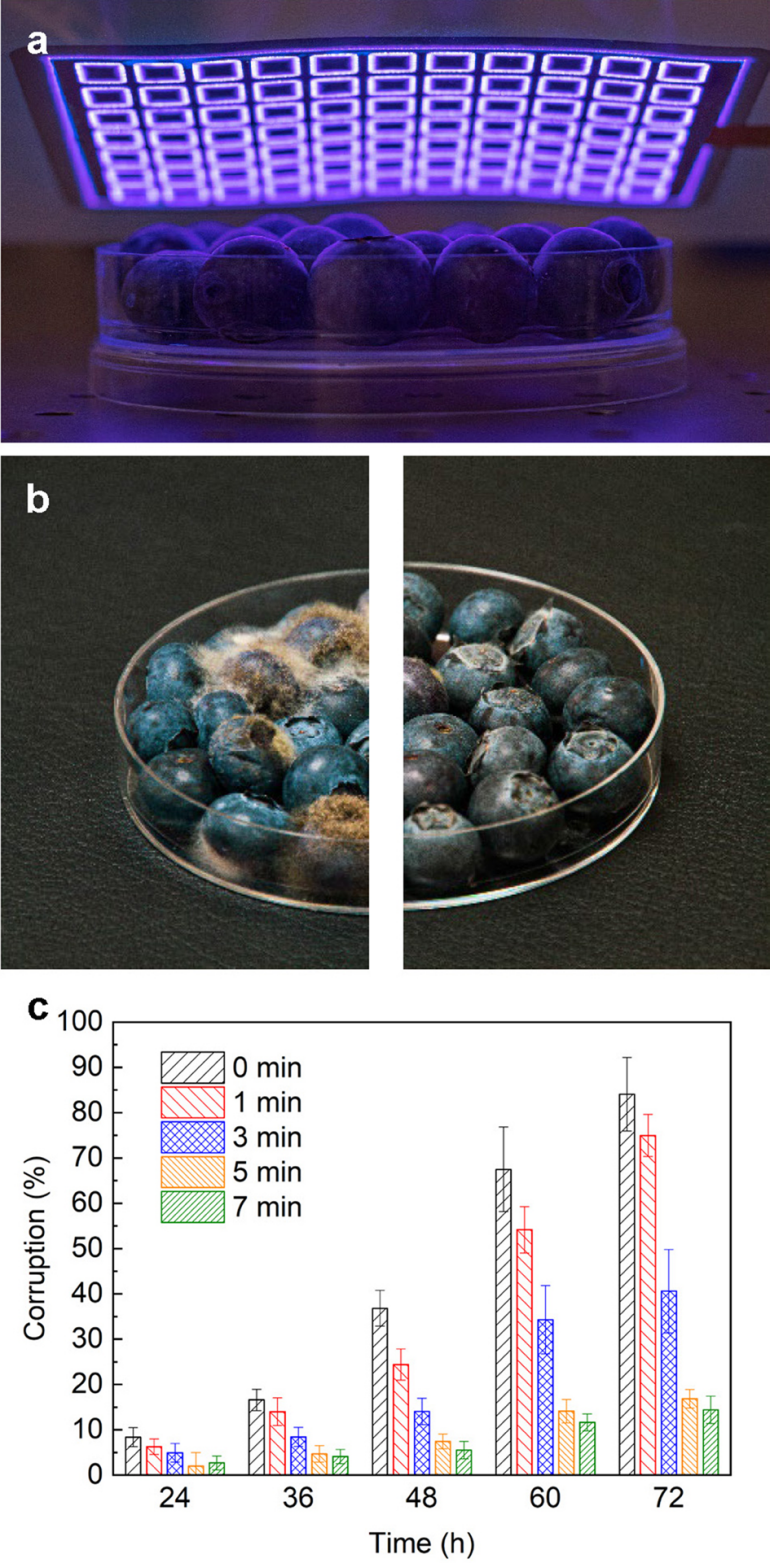
(**a**) In-package plasma treatment of blueberries. (**b**) Control and plasma-treated (5 min) blueberries after 72 h of storage. (**c**) Corruption rate of the blueberries according to the plasma treatment time. FXDBDs are driven by 15 W with 30 kHz bipolar square wave voltage.

As noted, the need for ‘new normal’ health technology has been reported; for example, such technology may be used with sterile packs, which are indispensable for food storage and the biomedicine field. Compared to conventional methods, plasma has become an emerging, green processing technology with many potential applications^5^. As a simple demonstration, we performed a feasibility test of FXDBD involving the shelf life extension of blueberries in a disposable packaging pouch for food storage (Figs. 6a,S1a). A simple plasma pouch consisting of a commercial plastic zipper bag and the proposed FXDBD source was fabricated. The FXDBD source was attached to the inner surface of a gas-tight food packaging pouch. Two 1 cm by 1 cm square sections of the zipper bag were replaced with a copper sheet for a connection between FXDBD and an external power supply while maintaining airtightness.

The photographs shown in Fig. 6b indicate that fungal growth was remarkably reduced in the blueberry samples treated with plasma. The fungus was distributed throughout the blueberry sample without plasma treatment, as shown in the left image, and 5 min plasma-treated samples were free from fungus. Figure 6c presents the corruption rate of blueberries based on the plasma treatment time and storage period. Corruption was judged based on the occurrence of fungal colonies. After 72 h of storage, white fungi grew in 84% of the control group, and the corruption rate decreased to 15% with 7 min plasma treatment. It is well known that the sterilization efficacy is proportional to the plasma treatment time with a constant concentration of active species such as O_3_^33^. However, the degree of corruption after plasma treatment was partly consistent with the timing of O_3_ and NOx production in the plasma pouch (Figure S1b); both concentrations sharply increased during the first 3 min and were plateaued, as a bactericidal effect appeared in the first few minutes of treatment and led to saturation conditions. Our results indicate that 5 min of plasma treatment in an FXDBD operated at 4 kW/m^2^ is enough to extend the lifetime of 30 blueberry samples. A more quantitative assessment about the reduction of pathogen and quality changes by FXDBD was done by separate research^34^. Considering the price side, the cost of manufacturing a set of plasma pouches in a laboratory environment is $0.6 each.

## Conclusion

This study reports the development of a flexible plasma source using an inkjet printing method, from manufacturing to characterization for durability, and a practical demonstration is presented. We introduce a proof-of-concept for the inkjet-printed FXDBD source. Inevitable stresses caused by plasma on the bare inkjet-printed electrode degrade the FXDBD source, limiting the operational life span and consequently its applicability. We clearly demonstrate that simple ways to make an inkjet-printed electrode more resistive include multiple inkjet printing and passivation. This plasma film, FXDBD, is operable in two distinct modes associated with RONS production in the power range of 25–40 W. The mode change from ozone-dominant to nitrogen oxide-dominant chemical production appeared with increasing input power. Finally, the developed FXDBD was applied to lengthen the shelf life of blueberries, with distinct fungal growth inhibition. Blueberries treated with plasma for at least 5 min inside a disposable pouch displayed an 80% decrease in the corruption rate compared to controls.

## Methods

### Quantifying stable gaseous chemical species

For characterizing plasma, an FXDBD source was installed in a gas-tight chamber (Figure S2). A stainless-steel cuboid chamber with dimensions of 150 mm × 150 mm × 100 mm was used, and the 100 mm × 100 mm FXDBD source was located therein. Two facing gas ports on the sidewalls of the chamber were installed for gas analysis. The copper sheet electrode was connected to the high-voltage power supply (TREK 10/10B-HS or FTLAB HPI500), and the inkjet-printed electrode was grounded. A 100 nF capacitor connected in series to the ground electrode was used as a reference capacitor to determine the Q–V Lissajous curve associated with the amount of electric power dissipated to plasma. The voltage across the 100 nF capacitor was measured using a 10:1 voltage probe (Tektronix P2100) combined with an oscilloscope (Keysight DSOX3034A), and the voltage across the FXDBD source was measured using a 1000:1 voltage probe (Tektronix P6015).

The reactive species produced inside the chamber were quantified by sampling gas through the ports. Prior to gas analysis, we verified that the use of a 1 m long polyurethane tube is reliable in gas analysis. Compared to the concentration measured when using stainless steel or Teflon tubes, little to no change in chemical concentrations occurred during gas transport from the chamber to the gas analyzers. Nitrogen oxides (NOx) and ozone (O_3_) concentrations were measured using an Eco Physics nCLD63 NOx analyzer and a 2BTechnology 106-M ozone analyzer, respectively. Note that dry synthetic air was supplied to the chamber at a 1.5 slpm flow rate, which was balanced with the sampling rate of the gas analyzer.

Before each experiment, the inside of the chamber was cleaned with ethyl alcohol, and each FXDBD source was a “one-off source” throughout the experiments. The temperature of the chamber was set at 30 °C using a hot plate (DAIHAN Scientific MSH-20D) throughout each experiment, and the gas temperature inside the chamber was determined using an optical thermometer (FISO FTI-10). The air altered by plasma was totally replaced by dry air (21% oxygen with balanced nitrogen, 0% relative humidity) after each experiment.

### Plasma-assisted blueberry storage

In order to exclude differences in harvest time and region between blueberry grains, blueberries harvested on the same date on the same farm were purchased and immediately stored at 7 °C. To exclude external contaminants, 100 g of blueberries at a time were inoculated with 500 mL of distilled water, vortexed for 1 min on a clean bench. This process was repeated for all blueberries purchased. After that, blueberries were divided into 50 g portions per single sample in sterilized Petri dishes. According to the plasma operation time, 5 samples for each experimental condition, a total of 25 samples were prepared. After placing the blueberry samples in the center of the pouch, the pouch air was purged with dry synthetic air. Plasma treatment experiments were conducted using 15 W with 30 kHz bipolar square wave voltage-driven FXDBDs, with the corresponding chemical behavior shown in Figure S1b for 0, 1, 3, 5, 7, and 9 min treatment times. Note that there was no difference in the appearance of the blueberries with and without plasma treatment. Treated samples were regularly monitored 12 h apart during storage at 25 °C in a 40% humidity environment without post-treatment measures. Korea Advanced Institute of Science and Technology (KAIST, Republic of Korea) approved the experiments, including any relevant details. The research facility follows the Standard Microbiological Practices for Biosafety Level 1 (BSL1) guideline.

### Energy-dispersive X-ray spectroscopy

Characterization of the inkjet-printed electrode was tested by using Quattro environmental scanning electron microscopy (ESEM) equipped with an EDS unit (Thermo Fisher Scientific) at X-ray energies lower than 10 keV. We observed that the initial mass ratio of electrode materials Ag, Al, Cl, O, and C were changed from 81.2%, 8.5%, 5.2%, 4.3%, and 0.8% to 70.7%, 13.6%, 2.3%, 11.5%, and 1.9%, respectively, due to 6 min plasma operation. Based on the measured mass ratio, atomic percents were estimated by considering the atomic number of each element.

## Supplementary Information

Supplementary Information.
