# Supplementary material for: Surface plasma with an inkjet-printed patterned electrode for low-temperature applications
Source: Sci Rep. 2021 Jun 9;11:12206. doi: 10.1038/s41598-021-91720-3 (PMC8190151; doi:10.1038/s41598-021-91720-3)
Supplement: Supplementary file 1 — Supplementary Information. [file 41598_2021_91720_MOESM1_ESM.pdf]

Supplementary material

Surface plasma with an inkjet-printed patterned electrode for low-temperature applications

Jinwoo Kim1, Sanghoo Park2, and Wonho Choe1, 3,*

[1]Department of Physics, Korea Advanced Institute of Science and Technology (KAIST), 291 Daehak-ro, Yuseong-gu, Daejeon 34141, Republic of Korea

[2]Institute of Plasma Technology, Korea Institute of Fusion Energy (KFE), 37 Dongjangsan-ro, Gunsan, Jeollabuk-do 54004, Republic of Korea

[3]Department of Nuclear and Quantum Engineering, KAIST, 291 Daehak-ro, Yuseong-gu, Daejeon 34141, Republic of Korea

*wchoe@kaist.ac.kr

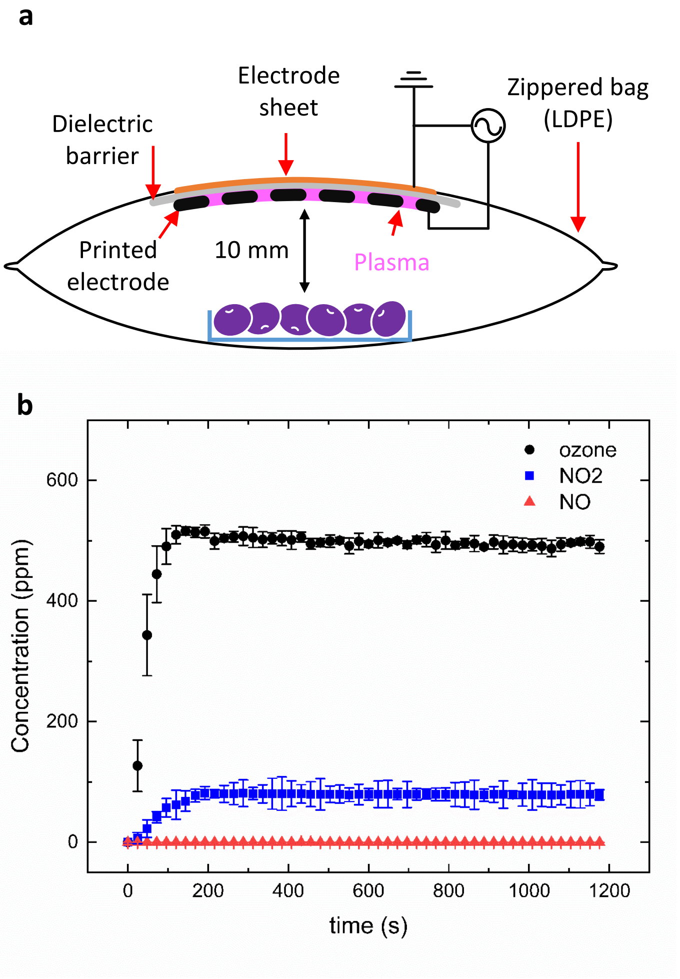

Figure S1. (a) Schematic representation of the feasibility of blueberry storage using a plasma pouch. (b) Concentration of active species generation in a plasma pouch under 15-W discharge conditions using 30 kHz bipolar square wave voltage source. 1.5 L/min of dry air was injected to balance the sampling volume of the measuring equipment. Data are shown as the mean and standard deviation from three independent measurements.

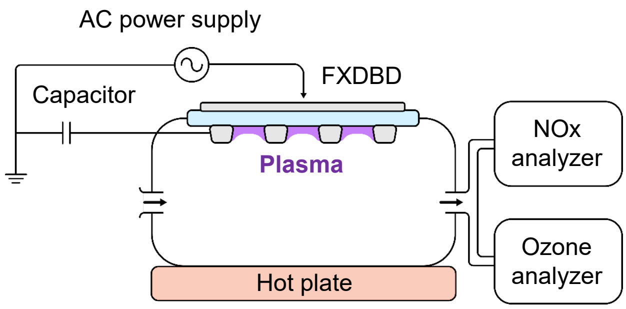

Figure S2. Experimental setup for characterizing FXDBD, including gas analysis and power measurements. The test chamber was maintained at atmospheric pressure with gas flowing to the analyzers.
